# Dual Effect of Exogenous Nitric Oxide on Neuronal Excitability in Rat Substantia Gelatinosa Neurons

**DOI:** 10.1155/2014/628531

**Published:** 2014-01-08

**Authors:** A-Reum Park, Hae In Lee, Dejidnorov Semjid, Do Kyung Kim, Sang Woo Chun

**Affiliations:** ^1^Department of Oral Physiology, College of Dentistry, Institute of Wonkwang Biomaterial and Implant, Wonkwang University, 344-2 Shinyong Dong, Iksan 570-749, Republic of Korea; ^2^Oral Biology Research Institute, Chosun University School of Dentistry, Gwangju 501-759, Republic of Korea

## Abstract

Nitric oxide (NO) is an important signaling molecule involved in nociceptive transmission. It can induce analgesic and hyperalgesic effects in the central nervous system. In this study, patch-clamp recording was used to investigate the effect of NO on neuronal excitability in substantia gelatinosa (SG) neurons of the spinal cord. Different concentrations of sodium nitroprusside (SNP; NO donor) induced a dual effect on the excitability of neuronal membrane: 1 mM of SNP evoked membrane hyperpolarization and an outward current, whereas 10 *µ*M induced depolarization of the membrane and an inward current. These effects were prevented by hemoglobin and 2-(4-carboxyphenyl)-4,4,5,5-tetramethylimidazoline-1-oxyl-3-oxide potassium salt (c-PTIO) (NO scavengers), phenyl *N*-tert-butylnitrone (PBN; nonspecific reactive oxygen species scavenger), and through inhibition of soluble guanylyl cyclase (sGC). Pretreatment with n-ethylmaleimide (NEM; thiol-alkylating agent) also decreased effects of both 1 mM and 10 *µ*M SNP, suggesting that these responses were mediated by direct S-nitrosylation. Charybdotoxin (CTX) and tetraethylammonium (TEA) (large-conductance Ca^2+^-activated K^+^ channel blockers) and glybenclamide (ATP-sensitive K^+^ channel blocker) decreased SNP-induced hyperpolarization. La^3+^ (nonspecific cation channel blocker), but not Cs^+^ (hyperpolarization-activated K^+^ channel blocker), blocked SNP-induced membrane depolarization. In conclusion, NO dually affects neuronal excitability in a concentration-dependent manner via modification of various K^+^ channels.

## 1. Introduction

Nitric oxide (NO) is a pivotal signaling molecule involved in many diverse developmental and physiological processes in the mammalian nervous system [1–3]. NO is biosynthesized from L-arginine by specific neuronal and non-neuronal forms of NO synthase [4, 5]. NO donors as well as endogenously produced NO play a role in many physiological processes, including smooth muscle relaxation, cellular proliferation, apoptosis, neurotransmitter release, and cell differentiation [6]. NO-induced effects are commonly mediated through the following processes: increased cGMP production upon activation of NO-sensitive soluble guanylyl cyclase (sGC), S-nitrosylation, tyrosine nitration, and NO interaction with superoxide (O_2_^•−^) to form peroxynitrite (ONOO^−^) [1, 7, 8].

Oxidative stress due to reactive oxygen species (ROS) such as O_2_^•−^, hydrogen peroxide (H_2_O_2_), NO, and ONOO^−^ interferes with normal cell function and can cause cell damage. Moreover, ROS is associated with chronic pain, particularly neuropathic and inflammatory pain [9, 10]. NO has a dual role in the regulation of pain processes; it can mediate a nociceptive or induce an antinociceptive effect. Some studies suggest that spinal NO is involved in the potentiation of nociception. For example, it has been demonstrated that nerve injury- or tissue inflammation-induced mechanical hypersensitivity is reduced in nNOS knockout mice and by intrathecal administration of nNOS inhibitors [11–13]. Furthermore, NO, produced in the NOS-containing spinal cord neurons, plays a pivotal role in chronic pain [14, 15].

In contrast, other studies have shown that administration of NO donors can induce antinociceptive effects. For example, L-arginine and 3-morpholinosydnonimine (SIN-1; NO donor), administered intracerebroventricularly to mice, cause antinociception [16]. Intraplantar injection of sodium nitroprusside (SNP), a substance which nonenzymatically releases NO, also causes antinociception in rats [17].

The substantia gelatinosa (SG) of the dorsal horn is the first site of synaptic transmission in the nociceptive pathway, and it is an area vital for the integration and modulation of the peripheral nociceptive input. Understanding neuronal excitability in this area is fundamental to enhance our knowledge on nociceptive neurotransmission. However, despite many reports on the importance of NO in nociceptive processing in the spinal cord, the effect of NO on the excitability of spinal cord dorsal horn neurons remains unclear. In this study, the effect of different concentrations of NO on the membrane potential of SG neurons was investigated using patch-clamp recordings from transverse slices of the spinal cord.

## 2. Materials and Methods

### 2.1. Spinal Cord Slice Preparation

Sprague-Dawley rats (14–18 days old) were first anesthetized with ether. The procedures were approved by the University of Wonkwang Committee on Ethics in the Care and Use of Laboratory Animals (WKU09-076). Lumbosacral laminectomy was performed following intraperitoneal administration of 25% urethane. The spinal cord at spinal level L1-S3 was removed and placed in a preoxygenated solution at 1-2°C. Transverse spinal slices, 350 *μ*m thick, were prepared using a vibroslicer (752M, Campden Instruments, Loughborough, UK) and incubated at 32°C for a recovery period of at least 1 h. Afterwards, slices were transferred to a recording chamber mounted on a upright microscope.

### 2.2. Solution and Drugs

The dissecting solution for the spinal cord slice preparation was composed of (in mM) 252 Sucrose, 2.5 KCl, 0.1 CaCl_2_, 2 MgCl_2_, 10 Glucose, 26 NaHCO_3_, and 1.25 NaH_2_PO_4_. The extracellular fluid used for the patch-clamp recording contained (in mM) 117 NaCl, 3.6 KCl, 2.5 CaCl_2_, 1.2 MgCl_2_, 1.2 NaH_2_PO_4_, 25 NaHCO_3_, and 11 glucose. It was continually aerated with 95% O_2_/5% CO_2_, which kept the pH at approximately 7.4. The pipette (internal) solution contained (in mM) 150 K-Glu, 10 Hepes, 5 KCl, 0.1 EGTA, 2 Mg-ATP, and 0.3 Na GTP. The pH was adjusted to 7.3 by KOH. 1H-[1,2,4]oxadiazole[4,3-*α*]quinoxaline-1-one (ODQ) and glibenclamide were dissolved in DMSO to prepare a stock solution. SNP, hemoglobin (Hb), 2-(4-carboxyphenyl)-4,4,5,5-tetramethylimidazoline-1-oxyl-3-oxide potassium salt (c-PTIO), phenyl *N*-tert-butylnitrone (PBN), ODQ, lanthanum chloride, cesium chloride, charybdotoxin (CTX), tetraethylammonium (TEA), glibenclamide, apamin, and n-ethylmaleimide (NEM) were obtained from Sigma-Aldrich (St. Louis, MO, USA).

### 2.3. Patch-Clamp Recording

Microelectrodes were prepared from capillary glass tubes (TW150-3, WPI, USA) using a microelectrode pipette puller (PP830, Narishige, Japan). Patch pipettes, filled with the pipette solutions, were used at a resistance ranging from 6 to 8 M*Ω*. The substantia gelatinosa of the spinal cord was viewed with an upright microscope (BX50WI, Olympus, Japan). Membrane potential and current were recorded using an Axopatch 200B (Axon Instruments, USA) amplifier that was connected to a computer using an A/D converter (Digidata 1322A, Axon Instruments, USA). Membrane potential recording and data analyses were performed using pClamp software (Version 9.0, Axon Instruments, USA). Generated currents were filtered with a low-pass 8-pole Bessel filter at 2 kHz. All experiments were performed at room temperature (22 ± 1°C).

### 2.4. Fluorescence Imaging

For detection of nitric oxide, spinal cord slices were incubated with 10 *μ*M of 4-amino-5-methylamino-2,7-difluorofluorescein diacetate (DAF-FM DA) for 30 minutes at 32°C. The slices were examined on an inverted fluorescence microscope (LSM 510, Carl Zeiss, Germany). Excitation wavelength was 488 nm, and emission was measured at 515 to 565 nm. A time series was used to record images every 30 s.

### 2.5. Data Analysis

Differences in drug effects were analyzed using independent *t*-test and were considered significant when *P* < 0.05. Data are expressed as mean ± SEM.

## 3. Results

### 3.1. Effects of SNP (1 mM and 10 *μ*M) on the Membrane Excitability in Substantia Gelatinosa Neurons of the Spinal Cord

**Figure 1:**
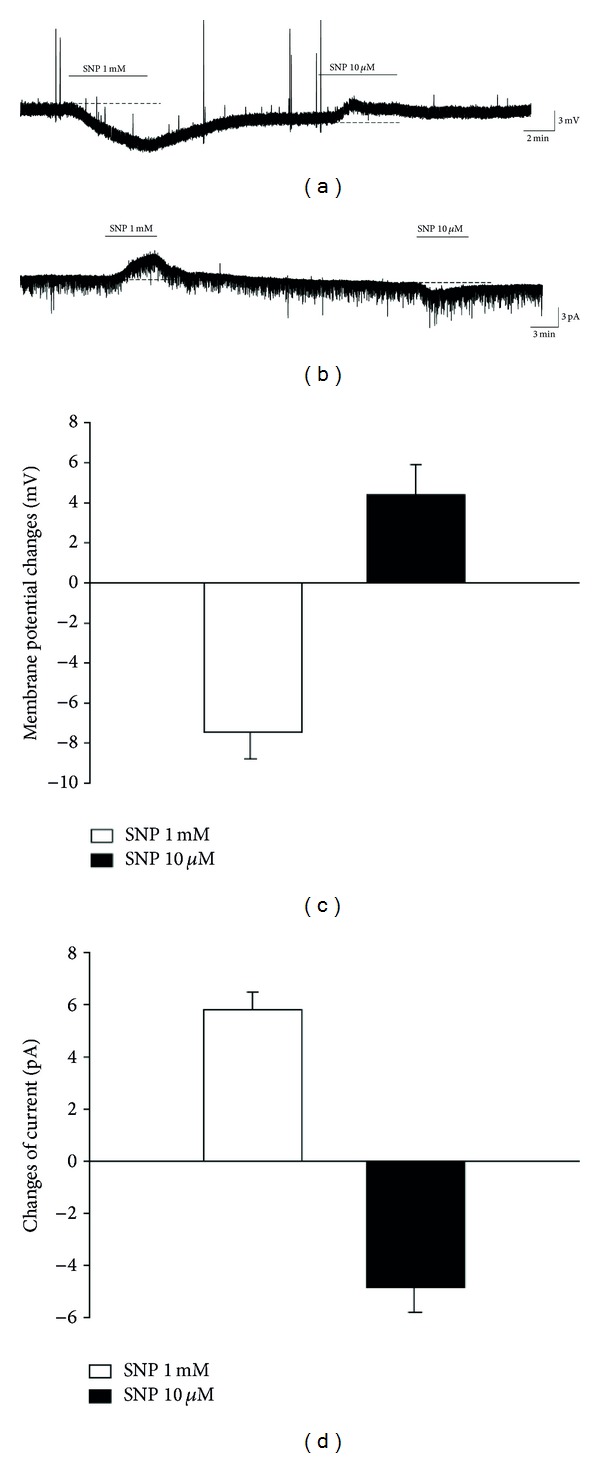
Effect of 1 mM and 10 *μ*M SNP on the membrane excitability in substantia gelatinosa (SG) neurons of the spinal cord. (a) Current-clamp recording of membrane potentials of SG neurons showing the dual effect of SNP. SNP (1 mM) induced membrane hyperpolarization, whereas SNP (10 *μ*M) elicited membrane depolarization. (b) Representative current traces of SG neurons recorded at a holding potential of −60 mV. SNP (1 mM) induced an outward current, whereas SNP (10 *μ*M) induced an inward current. (c) Bar graphs show the membrane potential changes evoked by different concentrations of SNP. (d) Bar graphs show the amplitude of current changes induced by different concentrations of SNP. Mean ± SEM.

During current-clamp recording, a high concentration of SNP (1 mM) induced membrane hyperpolarization (−7.5 ± 1.0 mV, *n* = 62), whereas a low concentration (10 *μ*M) induced membrane depolarization (4.4 ± 0.7 mV, *n* = 32) (Figures 1(a) and 1(c)). When voltage clamp recording was performed at a holding potential of −60 mV, SNP (1 mM) induced an outward current (5.7 ± 0.6 pA, *n* = 50), whereas SNP (10 *μ*M) induced an inward current (−4.8 ± 1.1 pA, *n* = 14) (Figures 1(b) and 1(d)). This suggests that SNP can elicit dual effects on the membrane excitability of SG neurons in a concentration-dependent manner.

### 3.2. Effects of NO Scavengers on SNP-Induced Membrane Potential Changes

**Figure 2:**
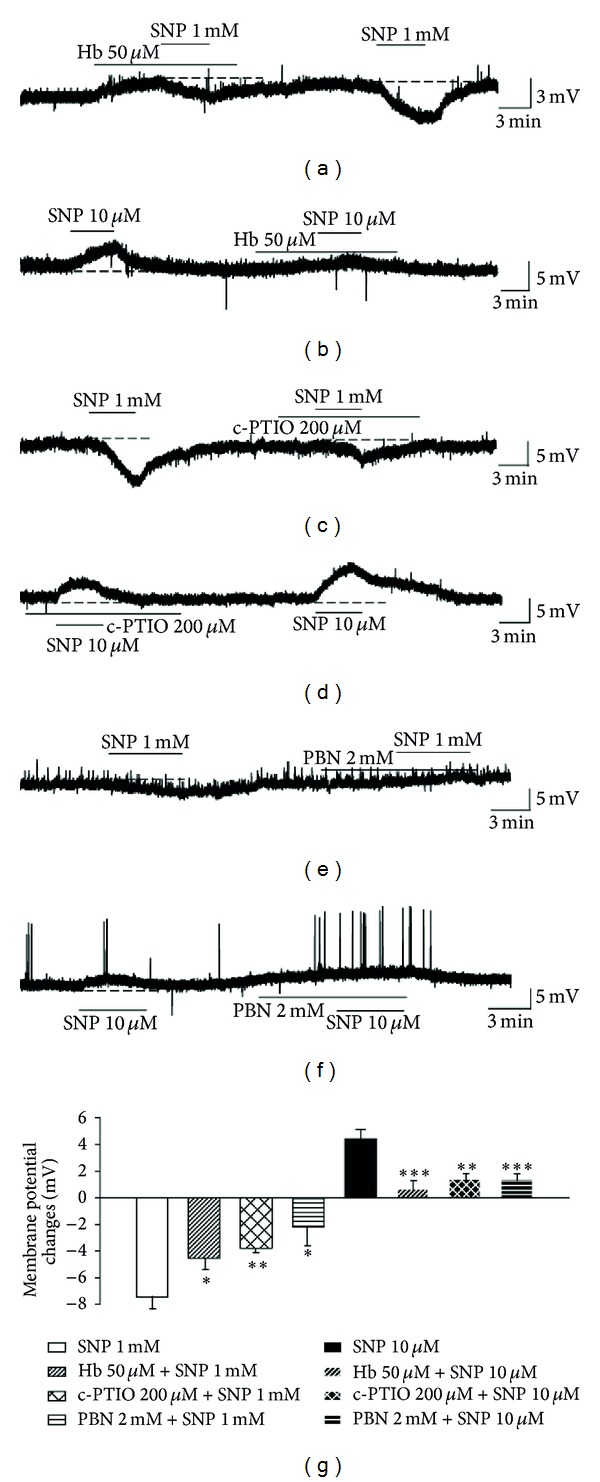
Effect of the NO scavengers on SNP-induced membrane potential changes. SNP (1 mM)-induced hyperpolarization was decreased by pretreatment with Hb (a) and c-PTIO (c). Hb (b) and c-PTIO (d) decreased SNP (10 *μ*M)-induced depolarization. (e) Pretreatment with PBN inhibited SNP (1 mM)-induced hyperpolarization. (f) PBN reduced SNP (10 *μ*M)-induced depolarization. (g) Summary of data obtained under the control condition of SNP-induced responses and pretreatment with Hb, c-PTIO, and PBN. *Values are significantly different from the control (SNP), based on independent *t*-test analysis (*P* < 0.05), **P < 0.01, ***P < 0.001. Mean ± SEM.

We next investigated the effects of NO scavengers to determine whether the SNP-induced changes in membrane potential were due to the release of NO from the donor. SNP (1 mM)-induced hyperpolarization is significantly reduced in the presence of the NO scavengers, Hb (50 *μ*M) (−4.5 ± 0.9 mV, *n* = 8, *P* < 0.05) and c-PTIO (200 *μ*M) (−3.7 ± 0.4 mV, *n* = 8, *P* < 0.01) (Figures 2(a), 2(c), and 2(g)). Furthermore, pretreatment with Hb (0.6 ± 0.6 mV, *n* = 6, *P* < 0.001) and c-PTIO (1.4 ± 0.5 mV, *n* = 5, *P* < 0.01) significantly inhibited SNP (10 *μ*M)-mediated depolarization (Figures 2(b), 2(d), and 2(g)). Pretreatment with PBN, the nonspecific ROS scavenger, significantly reduced SNP-induced hyperpolarization (−2.2 ± 1.6 mV, *n* = 5, *P* < 0.05) (Figures 2(e) and 2(g)) as well as SNP-induced depolarization (1.4 ± 0.4 mV, *n* = 5, *P* < 0.001) (Figures 2(f) and 2(g)). These results suggest that NO is released by SNP, which in turn induces the changes in membrane excitability of SG neurons.

### 3.3. Fluorescence Response of NO in DAF-FM DA-Loaded SG Neurons

**Figure 3:**
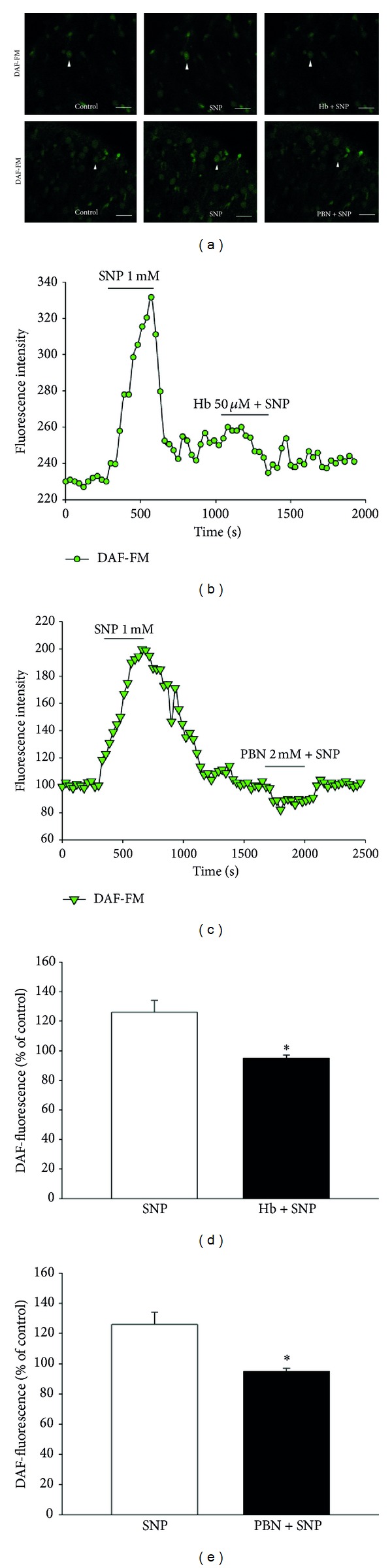
Fluorescence response of NO in DAF-FM DA-loaded spinal cord slices. (a) After addition of SNP (1 mM), fluorescence intensity increased. Hemoglobin (50 *μ*M) (upper) and PBN (2 mM) (lower) prevented the NO-induced fluorescence increase (scale bars: 50 *μ*m). ((b), (c)) Obtained images during the time series were shown for changes in fluorescence intensity within the regions of interest (ROI) (arrows indicate ROI). ((d), (e)) The results were quantitatively analyzed as percent units of DAF fluorescence of the control. *Values are significantly different from the control (SNP), based on independent *t*-test analysis (*P* < 0.05). Mean ± SEM.

The effect of SNP on NO production was determined using the cell-permeable fluorescent probe, DAF-FM DA. SNP is a donor of NO; thus, it can release NO, which then reacts with DAF-FM to produce fluorescence.  Figure 3 shows changes in intracellular fluorescence intensity over a time series of images taken every 30 s. Intracellular NO production was induced during SNP perfusion for 5 min. Increased NO production (128.0 ± 6.1%, *n* = 12) was inhibited by the NO scavenger, Hb (50 *μ*M) (92.0 ± 0.5%, *n* = 5, *P* < 0.05) (Figures 3(a), 3(b), and 3(d)), and the ROS scavenger, PBN (2 mM) (95.1 ± 2.1%, *n* = 7, *P* < 0.05) (Figures 3(a), 3(c), and 3(e)).

### 3.4. Involvement of Soluble Guanylyl Cyclase in the SNP-Induced Response

**Figure 4:**
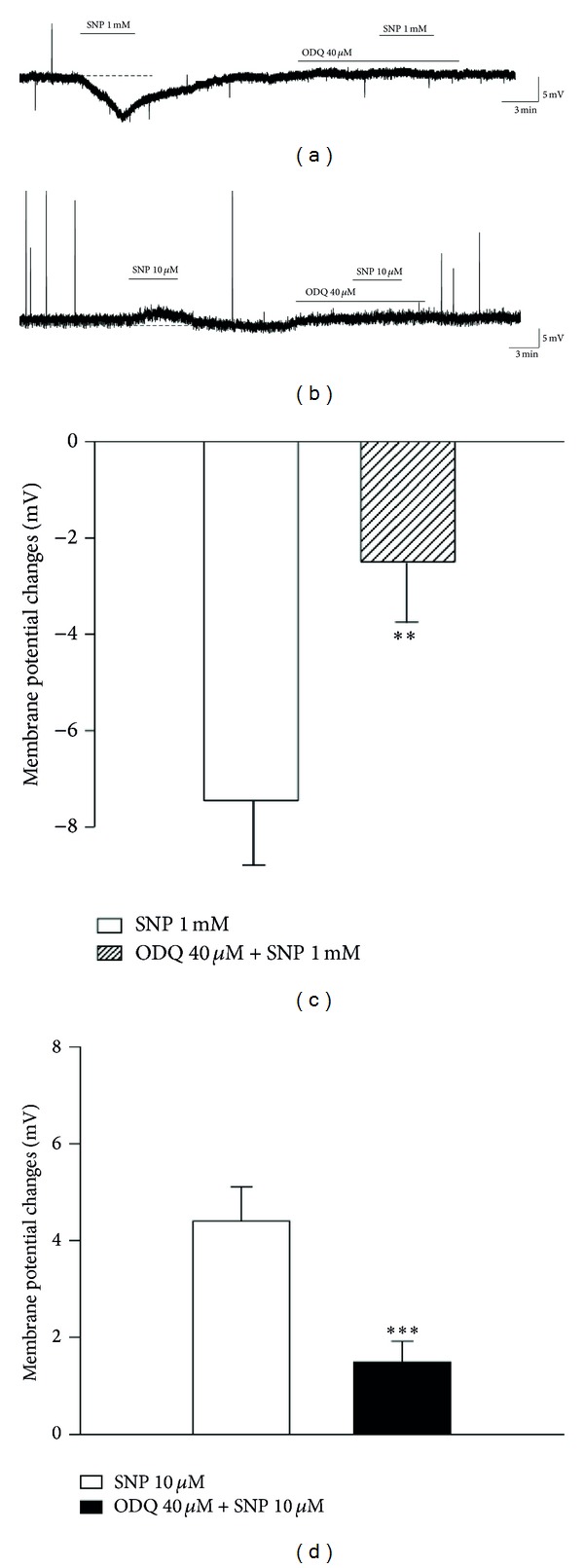
Soluble guanylyl cyclase is involved in the SNP-induced responses. (a) SNP (1 mM)-induced membrane hyperpolarization in SG neurons was blocked by ODQ (40 *μ*M). (b) Membrane depolarization by SNP (10 *μ*M) was inhibited by pretreated with ODQ. (c) Summary data obtained under the control condition of 1 mM SNP-induced hyperpolarization and pretreatment with ODQ. (d) Summary data obtained under the control condition of 10 *μ*M SNP-induced depolarization and pretreatment with ODQ. **Values are significantly different from the control (SNP), based on independent *t*-test analysis (*P* < 0.01), ***P < 0.001. Mean ± SEM.

NO has been shown to activate sGC, leading to an increase in cGMP levels. Thus, to determine whether the effect of SNP was mediated by the activation of sGC, ODQ (40 *μ*M), a selective sGC inhibitor, was used in the presence of both concentrations of SNP (1 mM and 10 *μ*M). Pretreatment with ODQ inhibited SNP (1 mM)-induced membrane hyperpolarization (−2.5 ± 0.9 mV, *n* = 6, *P* < 0.01) (Figures 4(a) and 4(c)) as well as SNP (10 *μ*M)-induced depolarization (1.5 ± 0.4 mV, *n* = 6, *P* < 0.001) (Figures 4(b) and 4(d)). These results suggest that the SNP-activated signaling pathway is dependent upon sGC.

### 3.5. Effect of a Thiol-Modifying Agent on the SNP-Induced Responses

**Figure 5:**
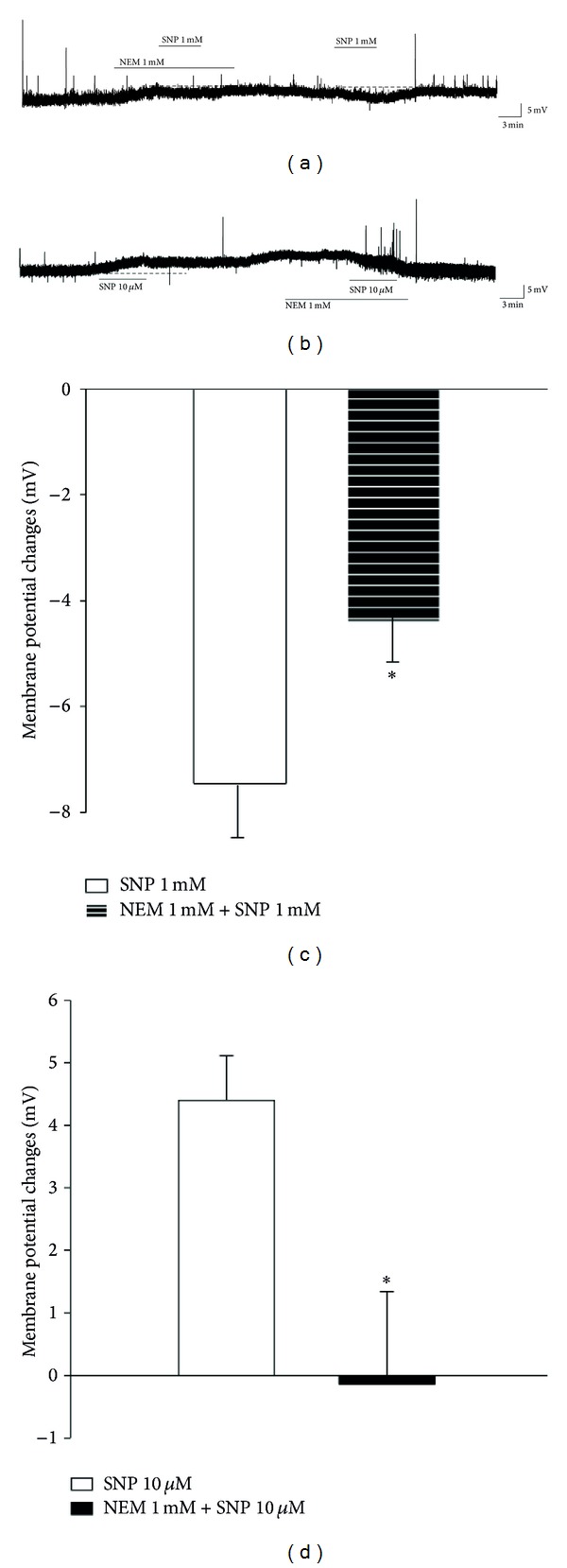
Effect of NEM, a thiol-modifying agent, on SNP-induced membrane potential changes. (a) Membrane hyperpolarization, induced by SNP (1 mM), was decreased by the presence of NEM. (b) SNP (10 *μ*M)-induced depolarization was decreased by the application of NEM. (c) Summary of data obtained under the control condition of SNP-induced hyperpolarization and pretreatment with NEM. (d) Summary of data obtained under the control condition of SNP-induced depolarization and pretreatment with NEM. *Values are significantly different from the control (SNP), based on independent *t*-test analysis (*P* < 0.05). Mean ± SEM.

A known alternative pathway for the biological effects of NO is the direct S-nitrosylation of critical cysteine thiol group(s) of target proteins [18]. To determine whether the SNP-evoked responses involved the direct modulation of membrane proteins by NO, we examined the effect of NEM, which blocks sulfhydryl groups, on SG neurons. Membrane hyperpolarization induced by SNP (1 mM) was significantly decreased by pretreatment with NEM (−4.4 ± 0.8 mV, *n* = 5, *P* < 0.05) (Figures 5(a) and 5(c)). Depolarization by SNP (10 *μ*M) was also significantly inhibited by the presence of NEM (−0.1 ± 1.4 mV, *n* = 7, *P* < 0.05) (Figures 5(b) and 5(d)). Similar results were observed for voltage clamp recordings. An inward current induced by SNP (10 *μ*M) and an outward current induced by SNP (1 mM) were inhibited by pretreatment with NEM (data not shown). These results indicate that SNP-induced responses are mediated via direct S-nitrosylation of channel protein.

### 3.6. Involvement of Various K^+^ Channels on SNP-Induced Membrane Hyperpolarization

**Figure 6:**
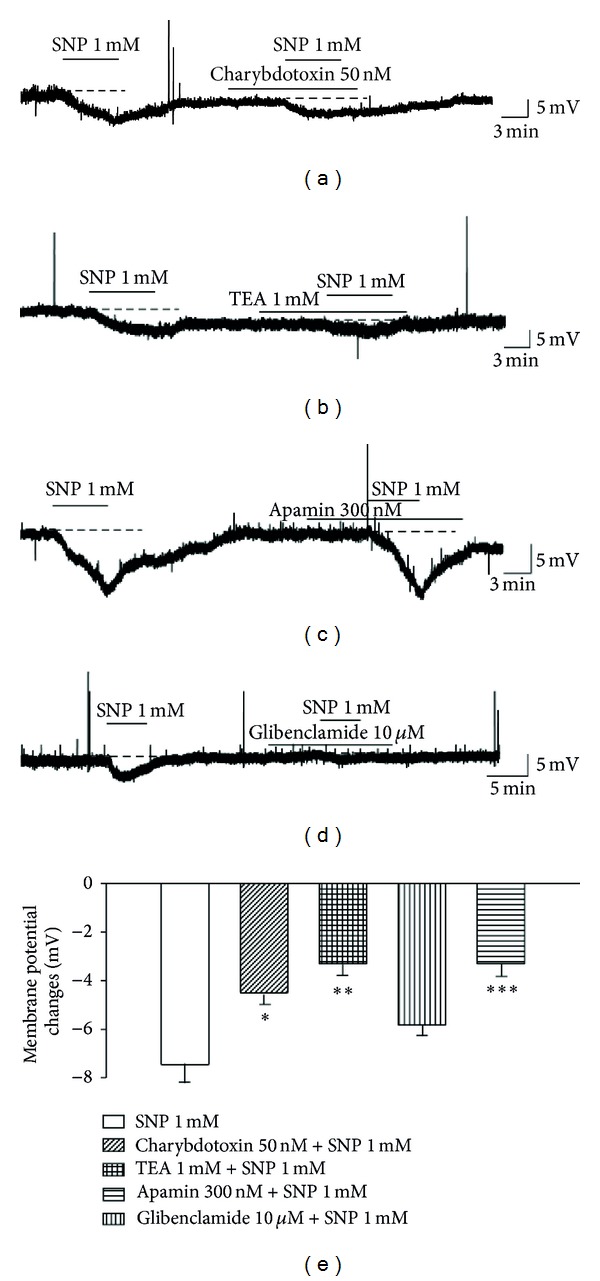
Involvement of various K^+^ channels in SNP-induced membrane hyperpolarization. ((a), (b)) Changes in membrane potential evoked by SNP (1 mM) were significantly inhibited by CTX and TEA, BK channel blockers. (c) Membrane hyperpolarization was not significantly inhibited by the presence of apamin, a SK channel blocker. (d) Membrane hyperpolarization was inhibited by application of glibenclamide, a K_ATP_ channel blocker. (e) Bar graphs show the membrane potential changes elicited by application of various K^+^ channel blockers. *Values are significantly different from the control (SNP), based on independent *t*-test analysis (*P* < 0.05), **P < 0.01, ***P < 0.001. Mean ± SEM.

Different mechanisms of NO-dependent effects have been reported in the literature, including the direct activation of K^+^ channels [1, 3, 19]. Therefore, we next determined the ion channels involved in the SNP-induced hyperpolarization. Significant inhibition of hyperpolarization was observed in the presence of CTX (−4.5 ± 0.7 mV, *n* = 6, *P* < 0.05) (Figures 6(a) and 6(e)) and TEA, large-conductance Ca^2+^-activated K^+^ (BK) channel blockers (−3.3 ± 0.7 mV, *n* = 10, *P* < 0.01) (Figures 6(b) and 6(e)). However, it was not significantly inhibited in the presence of apamin, small-conductance Ca^2+^-activated K^+^ (SK) channel blocker (−5.8 ± 0.7 mV, *n* = 6) (Figures 6(c) and 6(e)). In addition, membrane hyperpolarization was also significantly inhibited by application of glibenclamide, an ATP-sensitive K^+^ (K_ATP_) channel blocker (−3.3 ± 0.5 mV, *n* = 6, *P* < 0.001) (Figures 6(d) and 6(e)). These observations suggest that NO generated its effect through the activation of various K^+^ channels.

### 3.7. Involvement of a Nonspecific Cation Channel in Membrane Depolarization Induced by SNP

**Figure 7:**
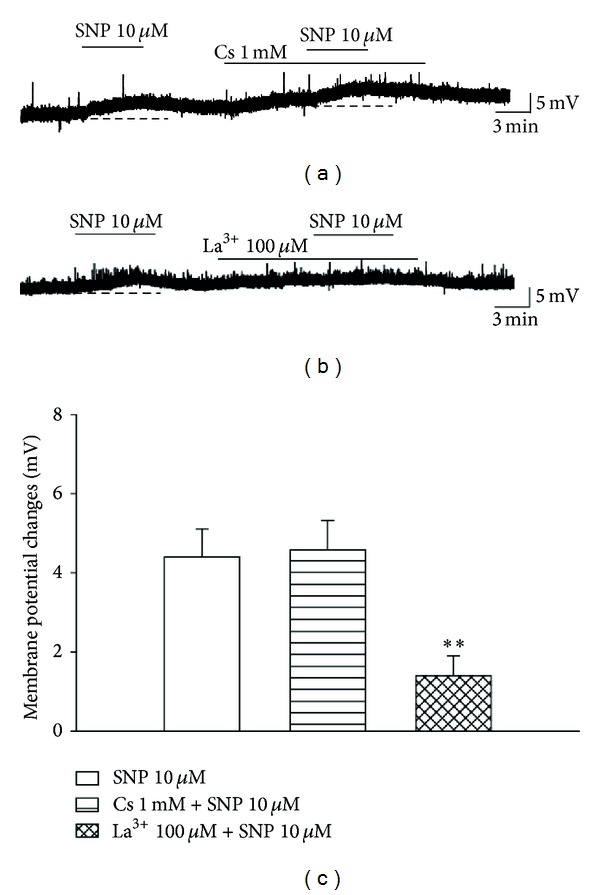
Involvement of a nonspecific cation channel in the membrane depolarization induced by SNP. (a) Membrane depolarization evoked by SNP (10 *μ*M) was not inhibited by the presence of 1 mM Cs^+^. (b) Depolarization evoked by SNP was significantly inhibited by La^3+^, a nonspecific cation channel blocker. (c) Bar graphs show the membrane potential changes induced by pretreatment with Cs^+^ and La^3+^. **Values are significantly different from the control (SNP), based on independent *t*-test analysis (*P* < 0.01). Mean ± SEM.

Recently, it was reported that SNP depolarizes the membrane potential of SG neurons and that this effect is inhibited by the presence of 1 mM Cs^+^ [20]. Based on this report, we tested whether SNP-induced depolarization was caused by the activation of hyperpolarization-activated K^+^ channel. Depolarization induced by a low concentration of SNP (10 *μ*M) was not inhibited by the presence of 1 mM Cs^+^ (4.5 ± 0.7 mV, *n* = 7) (Figures 7(a) and 7(c)). However, it was significantly blocked by the presence of a nonspecific cation channel blocker, lanthanum (1.4 ± 0.5 mV, *n* = 5, *P* < 0.01) (Figures 7(b) and 7(c)). These results suggest that depolarization induced by a low concentration of SNP (10 *μ*M) did not involve hyperpolarization-activated K^+^ channels but instead involved activation of a nonspecific cation channel.

## 4. Discussion

NO donors as well as endogenously produced NO exert various physiological effects, including smooth muscle relaxation, apoptosis, neurotransmitter release, and neurotoxicity [6]. NO is produced in the spinal dorsal horn neurons in response to extensive nociceptive input thereby contributing to central sensitization and persistent pain [21, 22].

Recently, a dual effect of NO on pain transmission was reported. Kawabata et al. [23] observed that NO induces a nociceptive or antinociceptive effect in a dose-dependent manner in mice. These authors demonstrated that injection of a low dose of L-arginine enhanced the nociceptive response, whereas administration of a high dose suppressed the nociceptive effect. In contrast, Li and Qi [24] demonstrated that intrathecal administration of low doses of L-arginine inhibited the nociceptive responses evoked by the intraplantar injection of formalin in rats, whereas high doses of the NO precursor increased this response. Furthermore, using a model of neuropathic pain in rats, Sousa and Prado [25] showed that intrathecal administration of 3-morpholinosydnonimine (SIN-1), a NO donor, produces a dual dose-dependent effect. These authors reported that low intrathecal doses of SIN-1 reduced the mechanical allodynia evoked by sciatic nerve ligation, whereas higher doses enhanced the allodynia or had no effect.

Pehl and Schmid [26] investigated the effects of different NO donors on spontaneously active neurons in the rat spinal cord using extracellular recording. They reported that NO causes direct excitation or inhibition of the electrical activity of spinal neurons. Discrepancies might be because of the differences regarding the doses of NO donors, the model used for pain evaluation, and experimental animal used in the studies [3]. Results, similar to those mentioned above, were also demonstrated in the present study, whereby application of different SNP concentrations produced a dual effect on the membrane potential of the SG neurons (Figure 1).

ROS such as O_2_^•−^, H_2_O_2_, NO, and ONOO^−^ are closely related to central sensitization [9, 10]. This study explored whether ROS are involved in the SNP-induced changes in neuronal excitability of SG neurons, produced by each concentration of SNP (1 mM or 10 *μ*M), by applying a strong ROS scavenger, PBN. Application of PBN significantly blocked the response evoked by both concentrations of SNP (Figure 2). It seems possible that NO can react with endogenously generated O_2_^•−^ to produce highly toxic ONOO^−^. ONOO^−^ has been proposed as a converged downstream molecule of O_2_^•−^ and NO in persistent pain conditions [12]. In this study, we did not use an ONOO^−^ decomposition catalyst to verify whether ONOO^−^ influences SNP-induced responses. Therefore, we cannot exclude the possibility that ONOO^−^ can modulate the excitability of SG neurons. However, Kim et al. [22] demonstrated that NO and O_2_^•−^ operate independently, while both are contributing to the same persistent pain.

Several fluorescent probes have been designed to measure NO in biological samples [27]. The most widely used and best characterized probes are 4,5-diaminofluorescein (DAF-2) and 4-amino-5-methylamino-2,7-difluorofluorescein (DAF-FM), both of which react with NO to form green fluorescent triazole products [28]. In this study, we confirmed the presence of SNP-induced intracellular NO production using DAF-FM. As shown in Figure 3, NO production was increased by addition of SNP in the spinal cord slices. Similar to our finding, it was previously reported that the fluorescence of DAF-FM increases in a dose- and time-dependent manner upon incubation with SNP [29]. The SNP-induced fluorescence increase observed in this study was reduced by Hb, a NO scavenger (Figures 3(a), 3(b), and 3(d)). The scavenging effect of Hb on NO has been demonstrated in several experiments [30, 31]. Moreover, we successfully used PBN, a ROS scavenger, to inhibit NO activity. Similar to a previous study [22], this result demonstrates that NO-induced fluorescence is prevented by PBN.

NO activates guanylate cyclase, which is responsible for an increase in intracellular levels of cGMP. Sousa and Prado [25] demonstrated that pretreatment with ODQ, a selective sGC inhibitor, practically abolishes the antinociceptive and pronociceptive effect mediated by an intrathecally applied NO donor. On the basis of their findings, in this study, ODQ was applied to each concentration of SNP to investigate the involvement of the NO-cGMP signaling pathway. Similar to previous reports, application of ODQ significantly blocked the response evoked by both concentrations of SNP (Figure 4). These findings demonstrate that SNP mediates its effect through a NO/sGC/cGMP pathway.

Besides activating the indirect cGMP-signaling pathway, NO can also directly modify channel proteins by S-nitrosylation [1, 18]. S-Nitrosylation is emerging as an important form of posttranslational modification of ion channels. It provides a route by which NO can regulate electrical activity without stimulating production of cGMP. Kawano et al. [18] reported that nitric oxide activates K_ATP_ channels in mammalian sensory neurons by direct S-nitrosylation. They showed that inhibition of sGC and PKG failed to block this activation by NO. In addition, they reported that NO activation of K_ATP_ currents is inhibited by thiol-alkylating agents, which demonstrates that S-nitrosylation is needed for NO action. In the present study, to determine whether SNP can directly modulate SG neurons through S-nitrosylation, NEM was applied as an S-nitrosylation blocker (Figure 5). The responses induced by both concentrations of SNP were significantly inhibited by NEM. These findings suggest that SNP mediates its effects via direct S-nitrosylation of membrane proteins in SG neurons.

K^+^ channel activation may be elicited by both NO and/or NO redox forms. Both PKG and S-nitrosylation enhance the activity of BK channels. In addition, cGMP modulates the activity of a delayed rectifier K^+^ channel and K_ATP_ channels through activation of PKG [1, 32–34]. To test whether NO activates K^+^ channels to induce changes in membrane potential, various K^+^ channel blockers were applied. Hyperpolarization evoked by SNP (1 mM) was significantly inhibited by pretreatment with CTX and TEA, BK channel blockers, and glibenclamide, a specific K_ATP_ channel blocker, but was not altered by pretreatment with apamin, a SK channel blocker (Figure 6). These findings indicate that NO-induced membrane hyperpolarization involves the activation of both BK, and K_ATP_ channel.

Kim et al. [20] observed that application of SNP (500 *μ*M) induced membrane depolarization in SG neurons and reported that this effect was elicited by a hyperpolarization-activated inward current. On the basis of this finding, we tested whether SNP (10 *μ*M)-induced depolarization was caused by the activation of a hyperpolarization-activated K^+^ channel by pretreating SG neurons with Cs^+^. However, membrane depolarization evoked by a low concentration of SNP was not affected by pretreatment with Cs^+^. Similar to our study, Sun et al. [35] demonstrated that peripheral ZD7288, a hyperpolarization-activated K^+^ channel blocker, blocked neuropathic pain while intrathecal administration of ZD7288 did not. Next, we used La^3+^, a nonspecific cation channel blocker, to block the membrane depolarization evoked by SNP. The membrane-depolarizing effect of SNP was significantly inhibited by pretreatment with La^3+^ (Figure 7). Recently, it was reported that NO donors could activate nonspecific cation channels including TRPV1 and TRPA1 by direct S-nitrosylation and indirect sGC/PKG pathway [8, 36, 37]. These results indicate that a nonspecific cation channel is involved in NO-related transmission of pain.

## 5. Conclusion

Substantia gelatinosa neurons in the dorsal horn are critical for mediating nociceptive signals. The dual effect of NO identified in SG neurons is important for the transmission of pain. The findings of this study suggest that NO elicits excitatory and inhibitory effects on SG neurons in a concentration-dependent manner via activation of various ion channels by direct S-nitrosylation and sGC activation.
